# C_2_ photosynthesis generates about 3-fold elevated leaf CO_2_ levels in the C_3_–C_4_ intermediate species *Flaveria pubescens*

**DOI:** 10.1093/jxb/eru239

**Published:** 2014-06-10

**Authors:** Olav Keerberg, Tiit Pärnik, Hiie Ivanova, Burgund Bassüner, Hermann Bauwe

**Affiliations:** ^1^Department of Plant Physiology, Institute of Agricultural and Environmental Sciences, Estonian University of Life Sciences, 51014 Tartu, Estonia; ^2^Center for Conservation and Sustainable Development, Missouri Botanical Garden, St. Louis, MO 63166-0299, USA; ^3^Department of Plant Physiology, University of Rostock, 18051 Rostock, Germany

**Keywords:** ^14^CO_2_ labelling, C_3_–C_4_ intermediate plants, carbon-concentrating mechanism, *Flaveria*, glycine decarboxylation, photorespiration, photosynthesis.

## Abstract

Formation of a photorespiration-based CO_2_-concentrating mechanism in C_3_–C_4_ intermediate plants is seen as a prerequisite for the evolution of C_4_ photosynthesis, but it is not known how efficient this mechanism is. Here, using *in vivo* Rubisco carboxylation-to-oxygenation ratios as a proxy to assess relative intraplastidial CO_2_ levels is suggested. Such ratios were determined for the C_3_–C_4_ intermediate species *Flaveria pubescens* compared with the closely related C_3_ plant *F. cronquistii* and the C_4_ plant *F. trinervia*. To this end, a model was developed to describe the major carbon fluxes and metabolite pools involved in photosynthetic–photorespiratory carbon metabolism and used quantitatively to evaluate the labelling kinetics during short-term ^14^CO_2_ incorporation. Our data suggest that the photorespiratory CO_2_ pump elevates the intraplastidial CO_2_ concentration about 3-fold in leaves of the C_3_–C_4_ intermediate species *F. pubescens* relative to the C_3_ species *F. cronquistii*.

## Summary

Photorespiration raises cellular CO_2_ levels about 3-fold in leaves of C_3_–C_4_ intermediate *Flaveria* species. This was shown by using ^14^C-based fluxomics to determine the Rubisco *in vivo* carboxylation-to-oxygenation ratios.

## Introduction

Land plants form three major classes characterized by specific modes of photosynthetic CO_2_ assimilation. In C_3_ plants, CO_2_ enters metabolism directly via ribulose 1,5-*bis*phosphate (RubP) carboxylase/oxygenase (Rubisco). In the mesophyll of C_4_ plant leaves and in CAM (crassulacean acid metabolism) plants, CO_2_ is initially fixed by phosphoenolpyruvate carboxylase. The resulting four-carbon (C_4_) compounds are decarboxylated in the Rubisco-containing bundle-sheath of C_4_ plants (Hatch and Slack, 1970) or become stored in the vacuoles of CAM plants for daytime decarboxylation and refixation of the released CO_2_ by Rubisco (Lüttge, 2004). Both modifications to the C_3_ mode of CO_2_ assimilation are adaptations to specific environmental conditions such as low CO_2_ or water availability. While C_4_ plants represent only about 3% of all land plant species, they dominate nearly all grasslands in the tropics, subtropics, and warm temperate zones (Sage, 2004). They also include highly productive crops, such as corn and sugar cane, and there is much interest to introduce yield-relevant features of C_4_ photosynthesis into C_3_ crops.

Given the ecological and agricultural significance of C_4_ plants, it is important to understand how they evolved and what were the crucial steps in this process. A number of studies have shown that the evolution of C_4_ photosynthesis was not a unique event but occurred at least 66 times during the past 35 million years (Sage, 2004; Sage *et al.*, 2012). Among these plant lineages, the small genus *Flaveria* (Yellowtops) has received particular attention because it includes species with CO_2_ assimilation modes ranging from C_3_ via a broad range of C_3_–C_4_ intermediate species to C_4_ (Powell, 1978; Apel and Maass, 1981; Ku *et al.*, 1983; Bauwe, 1984). Notably, extant *Flaveria* C_3_–C_4_ intermediate species represent true evolutionary intermediates between C_3_ and C_4_ photosynthesis (Kopriva *et al.*, 1996; McKown *et al.*, 2005). Major physiological features of such plants are low apparent photorespiration (Apel and Maass, 1981; Holaday *et al*., 1982, 1984) in combination with an enhanced refixation of photorespiratory CO_2_ (Holbrook *et al.*, 1985; Bauwe *et al.*, 1987) and high glycine accumulation (Holaday and Chollet, 1983, 1984).

Mechanistically, corresponding to the distribution of the photorespiratory enzyme glycine decarboxylase (GDC) in leaves of C_4_ plants (Ohnishi and Kanai, 1983), these specific characteristics are closely related to a confinement of GDC activity to the leaf bundle sheath (Hylton *et al.*, 1988; Moore *et al.*, 1988). Based on these and other data, it was hypothesized that C_3_–C_4_ intermediate species reduce apparent photorespiration by an efficient refixation of photorespired CO_2_ in the bundle sheath (Monson *et al.*, 1984; Edwards and Ku, 1987; Rawsthorne, 1992). This initial focus on the importance of CO_2_ refixation was later extended by the hypothesis that the confinement of glycine decarboxylase could result in a concentration of CO_2_ in the bundle sheath of C_3_–C_4_ intermediate plants (von Caemmerer, 1989; Monson and Rawsthorne, 2000). Today, such a mechanism, in which photorespiratory glycine serves as a vehicle to move ‘CO_2_’ from the mesophyll to the GDC-containing bundle sheath, is seen as a crucial step during the evolution of C_4_ photosynthesis (Bauwe, 2011; Sage *et al.*, 2012). In other words, the multiple evolution of C_4_ photosynthesis might have been triggered by and possibly even required the preceding presence of a much simpler CO_2_ concentration system than the C_4_ cycle, based on relatively small alterations to the high-flux photorespiratory glycine metabolism.

This hypothesis is now widely accepted and the genetic alterations necessary to restrict photorespiratory GDC activity to the bundle sheath are being unravelled (Wiludda *et al.*, 2012; Schulze *et al.*, 2013). On the other hand, it is not known how efficient this photorespiratory CO_2_ pump could be. Here, ^14^CO_2_ incorporation studies designed to obtain an estimate of the *in vivo* rates of the two Rubisco-catalysed reactions in the C_3_–C_4_ species *Flaveria pubescens* relative to the control C_3_ species *Flaveria cronquistii* are reported. The ratio of these reactions, carboxylation versus oxygenation of RuBP, is co-determined by kinetic parameters of Rubisco and by the CO_2_/O_2_ concentration ratio (Laing *et al.*, 1974; Peisker, 1974; Farquhar *et al.*, 1980). Hence, a higher *in vivo* carboxylation/oxygenation ratio in *F. pubescens* relative to a control C_3_ species would not only indicate an elevated CO_2_/O_2_ concentration ratio but also allow quantifying the efficiency of the photorespiratory CO_2_ pump.

## Materials and methods

### Plant growth and ^14^C labelling

*Flaveria cronquistii* A.M. Powell (C_3_), *Flaveria pubescens* Rydberg (C_3_–C_4_), and *Flaveria trinervia* (Spreng.) C. Mohr (C_4_) were grown in soil in a controlled environment chamber at 28/22 °C (day/night) and 250–300 µmol photons m^–2^ s^–1^ at a photoperiod of 16h. Fully expanded leaves excised from 40–60-d-old plants were fixed by thin wires in a frame positioned in a purpose-built fast-acting ^14^CO_2_ labelling device (Pärnik *et al.*, 1987). Leaves were pre-illuminated at 30 Pa ^12^CO_2_ and 210 kPa O_2_ for 10–15min at about 1200 µmol photons m^–2^ s^–1^ and 25 °C to ensure maximum stomata opening and achievement of the steady-state rate of photosynthesis. Plants were then exposed to ^14^CO_2_ (2000 MBq mmol^–1^) for 0.6, 1.2, 2.4, 5, 15, 60, 120, and 360 s at the same concentrations of CO_2_ and O_2_, temperature and light as applied during pre-illumination. At the given time points, within 0.1 s, the leaf samples were automatically transferred into boiling 80% ethanol. ^14^CO_2_ incorporation was linear over the whole experiment. All experiments were performed in triplicate (three individual plants in three consecutive days, resulting in three leaf samples per time-point for each species).

### Metabolite analysis

All leaf samples were individually extracted as described before (Värk *et al.*, 1968) with slight modifications. After 2min in boiling 80% ethanol, the samples were extracted for 15min at 86 °C with 5ml of 80% ethanol (twice) and 20% ethanol (once). All four ethanolic fractions were combined. The remaining samples were then further extracted for 15min at 86 °C with 5ml 96% ethanol acidified with 3 drops of 3 N HCl. The two extracts were separately (to avoid the hydrolysis of disaccharides) dried at 37 °C, individually re-dissolved in 5ml H_2_O each and cleared by centrifugation. The supernatants were combined, dried as above, and the metabolites re-dissolved in 1ml H_2_O. This final extract was used to determine total extractable radioactivity, radioactivity in amino acids (AAA 339 analyzer, Mikrotechna, Czech Republic), and other metabolites by using two-dimensional paper chromatography. Residual radioactivity in the fully extracted, dried, and triturated leaf samples was determined by using a non-aqueous scintillation cocktail. These analytical methods including the protocol used for starch analysis were described in more detail elsewhere (Keerberg *et al.*, 2011).

### Photosynthetic–photorespiratory gas exchange

Rates of net and true photosynthesis, photorespiratory CO_2_ evolution from the leaf, intracellular decarboxylation of early photosynthates, and rates of reassimilation of photorespiratory CO_2_ were determined during steady-state photosynthesis by using standard gas-exchange measurement techniques in combination with a radiogasometric method described before (Pärnik and Keerberg, 1995, 2007). In short, this method is based on the analysis of time curves of ^14^CO_2_ evolution from labelled photosynthates in leaves previously exposed to ^14^CO_2_. Photorespiration (210 kPa O_2_) and day respiration (15 kPa O_2_) were distinguished by measurement under different O_2_ concentrations. Re-fixation ratios (*D*) of photorespiratory CO_2_ were calculated from ^14^CO_2_ evolution at the very high concentration of 3 kPa ^12^CO_2_, where re-fixation of ^14^CO_2_ evolved inside the cell is close to zero, relative to ^14^CO_2_ evolution at air levels of ^12^CO_2_.

### Modelling and data analysis

From the radioactivity values for individual metabolites in combination with the specific radioactivity of the ^14^CO_2_ fed to leaves, the amounts of carbon incorporated at the selected time points were calculated and plotted against the duration of feeding with ^14^CO_2_. The amounts of carbon fixed in individual compounds were expressed in absolute (µmol C m^–2^) and relative (per cent of total carbon fixed) units. These experimental labelling curves contain the information about rates of all relevant carbon fluxes and corresponding metabolite pool sizes.

**Fig. 1.**
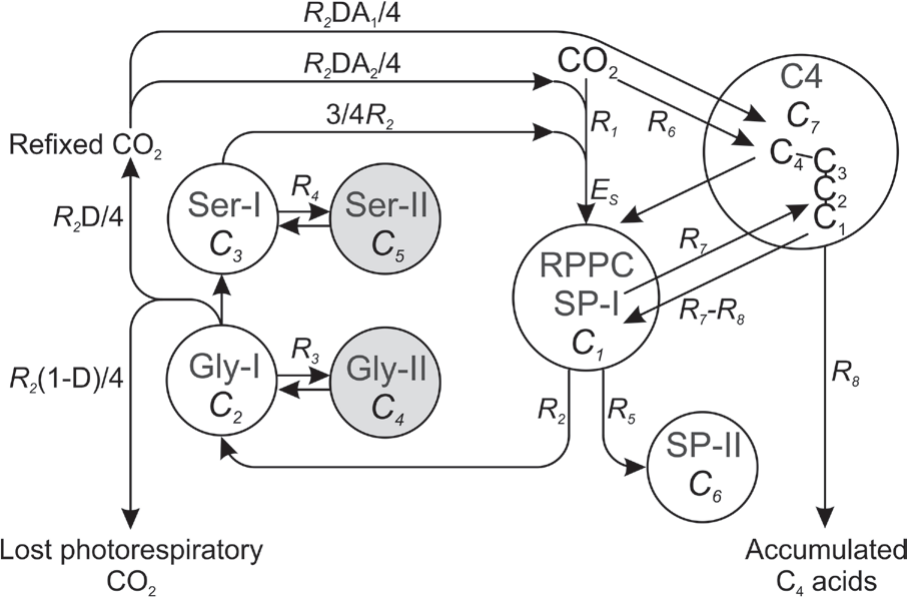
Model of major photosynthetic-photorespiratory carbon fluxes in *Flaveria* including the reductive pentose phosphate cycle (RPPC) with the attached photorespiratory pathway and the C_4_ photosynthetic pathway. *R*_1_, rate of CO_2_ fixation in RPPC; *R*_2_, rate of carbon flux through the glycolate cycle; *R*_3_, rate of carbon exchange between different pools of glycine; *R*_4_, rate of carbon exchange between different pools of serine; *R*_5_, rate of transport of sugar phosphates out of the RPPC; *R*_6_, rate of CO_2_ fixation by the C_4_ pathway; *R*_7_, rate of carbon flux from RPPC into ‘C_3_ skeletons’ of C_4_ acids, *R*_8_, rate of accumulation of C_4_ acids; *C*_1_, total pool of sugar phosphates in the RPPC; *C*_2_, active pool of the glycine branch of the photorespiratory pathway; *C*_3_, active pool of the serine branch of the photorespiratory pathway; *C*_4_ and *C*_5_, corresponding non-photorespiratory metabolite pools; *C*_6_, extra-cyclic pool of sugar phosphates; *C*_7_, total pool of C_4_ acids. *D* (reassimilation coefficient) describes the fraction of refixed relative to total photorespiratory CO_2_. *A*_1_ and *A*_2_ are the partition coefficients describing the relative contributions of the RPPC and the C_4_ pathway to refixation of photorespiratory CO_2_. Note that Gly-I and Ser-I also include all other metabolites from the respective branches of the photorespiratory pathway. Gly-II and Ser-II represent less mobile (cytosolic, plastidial, vacuolar) pools of these metabolites.

To extract this information on *Flaveria* photosynthetic–photorespiratory metabolism, the model shown in Fig. 1 was used. The model allows CO_2_ incorporation into the reductive pentose phosphate cycle (RPPC) either directly with rate *R*_1_ or via the C_4_ cycle with rate *R*_6_. Total carbon flux through the photorespiratory cycle is denoted *R*_2_. R_5_ is the export rate of phosphorylated sugars into other pathways, for example, sucrose biosynthesis. *R*_7_ denotes the rate of carbon efflux from the RPPC to the C_3_ skeleton of C_4_ acids, while *R*_8_ describes the rate of accumulation of C_4_-acids. In order to simplify calculations, metabolites were grouped into four pools: (i) pool ‘SP’ with sugar phosphates plus 3-phosphoglycerate, (ii) pool ‘Gly’ with metabolites of the two-carbon branch of the photorespiratory cycle, (iii) pool ‘Ser’ with metabolites of the three-carbon branch of the photorespiratory cycle, and (iv) pool ‘*C*_4_’ with malate and aspartate. Each of these four pools comprises two metabolic sub-pools with different labelling kinetics, for example, photorespiratory pools with rapid turnover in peroxisomes and mitochondria (Gly-I and Ser-I with pools *C*_2_ and *C*_3_, respectively) or less mobility in the cytosol and chloroplasts (Gly-II and Ser-II with pools *C*_4_ and *C*_5_, respectively). At steady-state photosynthesis, these pools are in diffusional equilibrium with exchange rates *R*_3_ and *R*_4_, respectively. At the glycine-into-serine conversion step, one molecule of CO_2_ is released per serine molecule formed, corresponding to a glycine decarboxylation rate of *R*_2_/4. The resulting CO_2_ is re-fixed in the RPPC or the C_4_ cycle or escapes from the leaf. The extent of re-fixation is described by the re-fixation coefficient *D*, which was experimentally determined as described above.

Formally, the metabolic model is described by the four analytical functions shown as equations 1–4, one for each major metabolite pool (similar to Keerberg *et al.*, 2011). To determine individual pool sizes *C*_i_ and carbon fluxes *R*_i_, the experimental values of the radioactivity of sugar phosphates, metabolites of the glycine and serine branches of the photorespiratory pathway, and of C_4_-acids were simultaneously fitted to these functions by multi-component non-linear regression analysis. These functions also consider the time-dependent dilution of the applied tracer CO_2_ by unlabelled photorespiratory CO_2_, which is important particularly at the start of tracer feeding under steady-state photosynthesis. A more detailed explanation of these functions is provided in the Supplementary data at *JXB* online.

## Results and discussion

The analysis of *in vivo* Rubisco carboxylation and oxygenation rates is not trivial. Potentially, such data can be extracted from gas exchange experiments (Pärnik and Keerberg, 1995), but this approach is biased by limited knowledge of the internal diffusion pathways for CO_2_ and O_2_. Bias becomes even stronger at a varying intercellular distribution of photosynthetic tasks, such as the operation of CO_2_-concentrating mechanisms. Assuming that there is no large variation in the plastidial O_2_ concentrations (Tolbert *et al.*, 1995), it should be possible approximately to assess the efficiency of the photorespiratory CO_2_ pump in C_3_–C_4_ intermediate plants by the quantification of carbon fluxes through the individual routes of the photosynthetic–photorespiratory biochemical network. Speed and complexity of the biochemical processes involved require fast and, consequently, sensitive labelling techniques using ^14^CO_2_ as a tracer in combination with model-based data analysis.

For our study, three *Flaveria* species were used, *F. cronquistii* (C_3_), *F. pubescens* (C_3_–C_4_ intermediate), and *F. trinervia* (C_4_). These species have previously been examined for their photosynthetic types (Apel and Maass, 1981; Ku *et al.*, 1983; Rumpho *et al.*, 1984), kinetic properties of Rubisco (Bauwe, 1984; Wessinger *et al.*, 1989; Kubien *et al.*, 2008), and phylogenetic position within the genus (Powell, 1978; Kopriva *et al.*, 1996; McKown *et al.*, 2005). These studies include the observation (Bassüner *et al.*, 1984; Monson *et al.*, 1986) that C_3_–C_4_ intermediate *Flaveria* species fix a small fraction of CO_2_ via the C_4_ pathway (*R*_6_ in the model shown in Fig. 1) while most of the CO_2_ enters metabolism directly via the RPPC (*R*_1_ in Fig. 1). It was not our intention to perform a comprehensive re-analysis of photosynthetic–photorespiratory carbon metabolism of these species. Instead, we wanted to focus on the quantification of key fluxes including control data confirming adequate fidelity of our approach.

Building upon previous studies (Pärnik *et al.*, 1987; Keerberg *et al.*, 2011), the model schematically shown in Fig. 1 was developed which embraces, in a generalized form, all the relevant information that is necessary to determine Rubisco carboxylation/oxygenation ratios *in vivo*. It considers time- and flux-dependent changes in the tracer’s specific radioactivity at all nodes of the network and allows the separation of high- and low-turnover pools of key metabolites of photosynthetic CO_2_ and photorespiratory O_2_ fixation. In order to simplify the model and make it as robust as possible, the metabolically related metabolites of the four major pathways were combined into four pools, each of which is described by a labelling function *P*(*t*,*C*_i_,*R*_i_) shown as equations 1–4.

$$P\left(SP\right)=S_{\text{S}}\left[C_{\text{1}}E_{\text{A}}\left(t,C_{\text{1}}R_{\text{S}}\right)+C_{\text{6}}E_{\text{I}}\left(t,C_{\text{1}},C_{\text{6}},R_{\text{5}}\right)\right]$$ (1)

$$P\left(Gly\right)=S_{\text{S}}\left[C_{\text{2}}E_{\text{l}}\left(t,V_{\text{1}},C_{\text{2}},R_{\text{2}}\right)+C_{\text{4}}E_{\text{l}}\left(t,V_{\text{2}},C_{\text{4}},R_{\text{3}}\right)\right]$$ (2)

$$\begin{array}{l} P\left(Ser\right)=S_{\text{S}}[C_{\text{3}}E_{\text{l}}\left(t,V_{\text{4}},C_{\text{3}},0.\text{75}\left(R_{\text{2}}-R_{\text{3}}\right)\right)\\ \;+C_{\text{5}}E_{\text{l}}\left(t,V_{\text{6}},C_{\text{5}},R_{\text{4}}\right)+V_{\text{6}}E_{\text{l}}\left(t,V_{\text{3}},V_{\text{6}},0.\text{75}R_{\text{3}}\right)] \end{array}$$ (3)

$$\begin{array}{l} P(C_{\text{4}})=0.\text{25}S_{\text{C}}C_{\text{7}}E_{\text{A}}\left(t,0.\text{25}C_{\text{7}},R_{\text{c}}\right)\\ \;+S_{\text{S}}[0.\text{75}C_{\text{7}}E_{\text{l}}\left(t,V_{\text{7}},0.\text{75}C_{\text{7}},R_{\text{7}}\right)\\ \;+E_{\text{E}}\left(t,V_{\text{8}},0.\text{25}R_{\text{8}}\right)+E_{\text{E}}\left(t,V_{\text{9}},0.\text{75}R_{\text{8}}\right)] \end{array}$$ (4)

Essentially, these four functions describe the time dependence of the radioactivity *P* incorporated under steady-state conditions into each of the four major model components sugar phosphates plus 3-phosphoglycerate [equation (1); SP-I plus SP-II], the glycine branch [equation (2); Gly-I plus Gly-II] and the serine branch [equation (3); Ser-I plus Ser-II] of the photorespiratory pathway, and the C_4_ pathway [equation (4); *C*_4_]. *S*_S_ and *S*_C_ are time-dependent functions that describe changes in the specific radioactivity of CO_2_ fixed in the RPPC and the C_4_ pathways, respectively. Functions *P(SP), P(Gly), P(Ser)*, and *P(C*_4_) were simultaneously fitted to experimental data points collected over a time scale from 0.6 to 360 s during steady-state photosynthesis. Quantitative values for carbon fluxes *Ri* between the sub-pools directly involved in photosynthetic CO_2_ fixation and photorespiration, for example, from SP-I (pool size *C*_1_) via Gly-I (pool size *C*_2_) to Ser-I (pool size *C*_3_), were calculated by multi-component non-linear regression analysis.

**Fig. 2.**
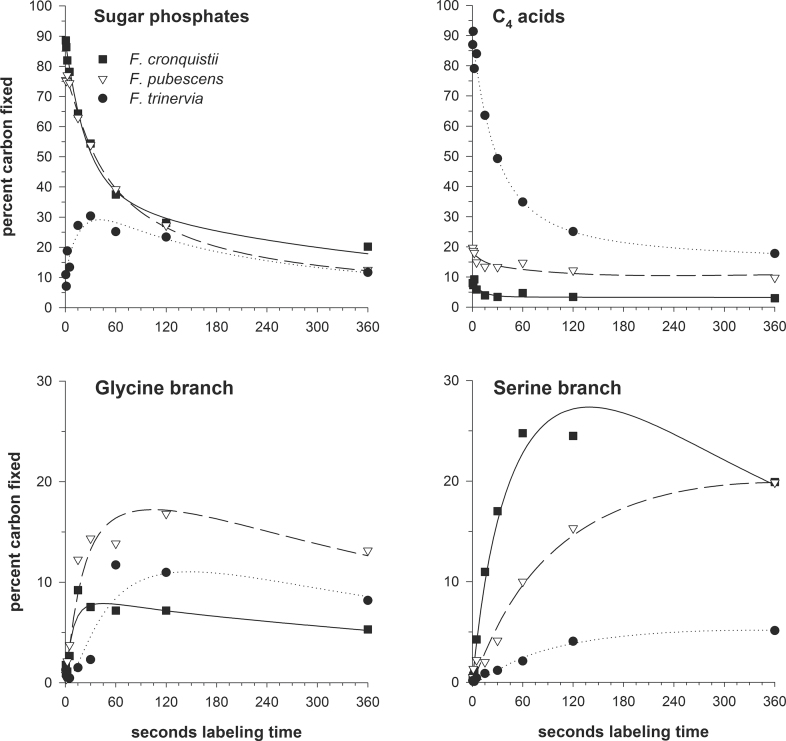
Time-courses of CO_2_ incorporation into sugar phosphates, C_4_ acids, and intermediates of the two branches of the photorespiratory pathway. Shown are time-courses relative to true photosynthesis, which was set to 100% for easier comparison. Symbols represent mean values from three data points (biological replicates). Solid (*F. cronquistii*), dashed (*F. pubescens*), and dotted lines (*F. trinervia*) are best fits to the labelling functions (Equations 1–4) and were calculated by multi-component non-linear regression analysis.

Figure 2 demonstrates that the model approximations for all four major metabolite pools represented by the model fit very well to the experimental data points. This includes initial CO_2_ fixation by the C_4_ pathway in *F. trinervia* in combination with final refixation of CO_2_ released from C_4_ acids by the RPPC as well as the ‘glycine anomaly’ of the C_3_–C_4_ intermediate plant *F. pubescens*. As mentioned in the Introduction, the specific alterations to glycine metabolism of C_3_–C_4_ intermediate plants are due to a specific distribution of photorespiratory GDC activity (Rawsthorne, 1992), which represents the enzymatic backbone of the photorespiratory CO_2_ pump.

**Fig. 3.**
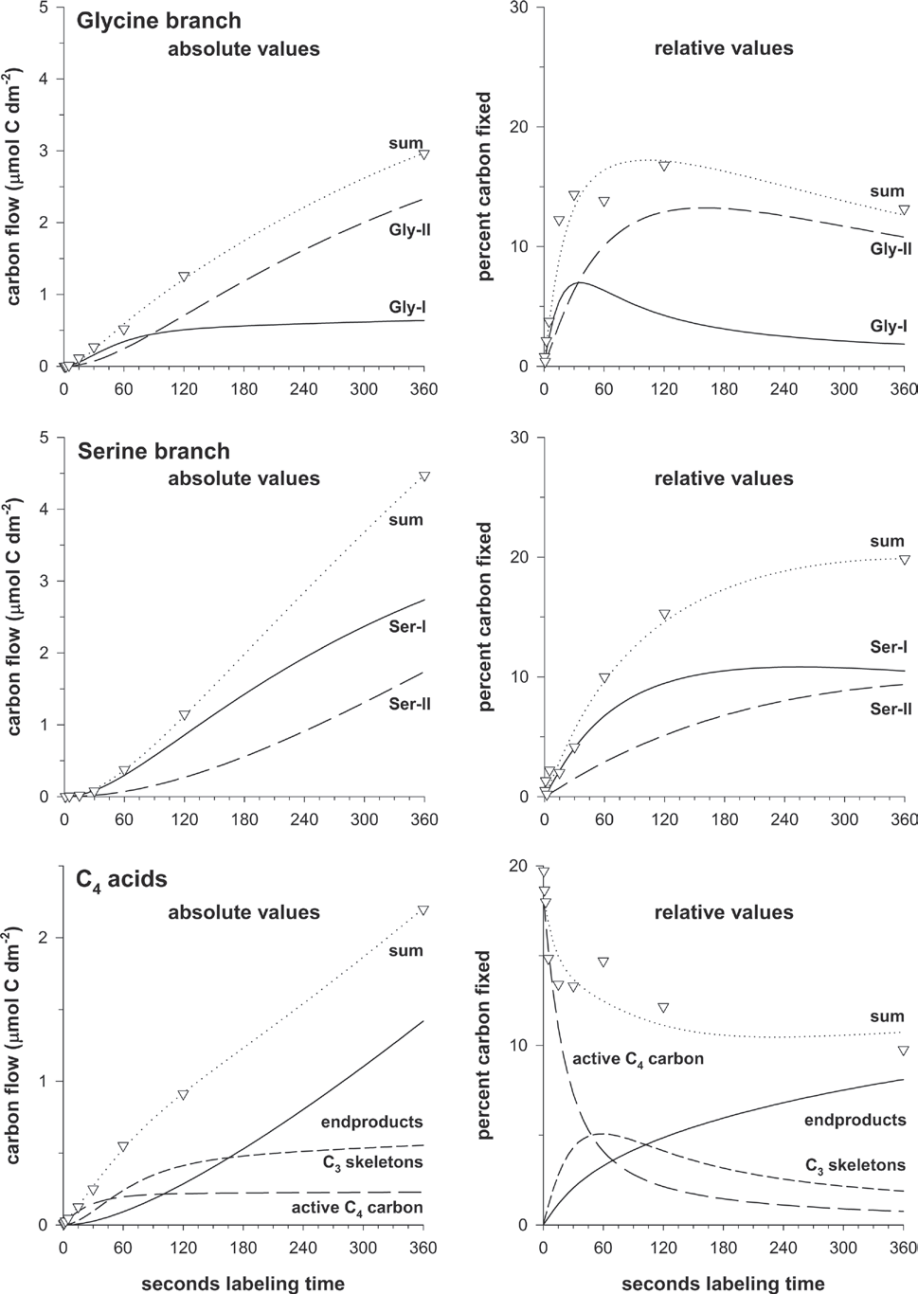
Examples for the model-based separation of fast- and slow-turnover pools in the ‘Gly’ and ‘Ser’ branches of the photorespiratory pathway and for primary versus secondary labelling and accumulation of C_4_ acids. All data are for *F. pubescens*.

Another apparent feature is the overlap of primary and secondary labelling kinetics, which is best seen with the C_4_ acids but also within the glycine and serine branches of the photorespiratory pathway (Keerberg *et al.*, 2011). In the case of the C_4_ acids, the complex labelling kinetics results from direct CO_2_ fixation (*R*_6_ in Fig. 1), secondary labelling of carbons 1–3 by the synthesis of phosphoenolpyruvate from RPPC intermediates (via phosphoglycerate mutase and enolase; *R*_7_), and export as a metabolically less mobile pool (probably to the vacuole; *R*_8_). Also, two metabolic pools with different labelling kinetics exist in both branches of the photorespiratory pathway. This is because one fraction each (Gly-II and Ser-II with pools *C*_4_ and *C*_5_, respectively) is present in cellular compartments that do not directly contribute to photorespiratory reactions. These fractions show a lower turnover than the photorespiratory most active pools (Gly-I and Ser-I with pools *C*_2_ and *C*_3_, respectively). At steady-state photosynthesis, the pools equilibrate pairwise with exchange rates *R*_3_ and *R*_4_. To consider such effects, and specifically calculate fluxes between metabolite pools directly involved in CO_2_ fixation and photorespiration, the model allows overlapping pools with different labelling kinetics to be separated by component analysis. Figure 3 provides examples of how the sequestration of metabolites into different pools was quantified and how the separation of primary and secondary labelling was achieved in the case of *F. pubescens*. The example data display carbon incorporation into high- (Gly-I and Ser-I) and low-turnover (Gly-II and Ser-II) pools within the glycine and serine branches of the photorespiratory pathway. They also demonstrate the quantitative separation of the ‘active’ *C*_4_ carbon pool of C_4_ acids from label appearing in carbon atoms 1–3 and in C_4_ acids exported to the vacuole. Collectively, these data show that the chosen model is an adequate tool for the calculation of fluxes through the major routes of photosynthetic CO_2_ fixation from quantitative ^14^CO_2_ labelling data.

The relevant fluxes are summarized in Table 1 and complemented by results from radiogasometric measurements performed in parallel with the same set of plants. These independent data show rates of true photosynthesis, total decarboxylation, and photorespiratory CO_2_ evolution. They allowed calculating the extent to which photorespiratory CO_2_ is re-fixed.

**Table 1.** Carbon fluxes in photosynthetic–photorespiratory carbon metabolism of *Flaveria* speciesValues marked with an asterisk represent means ±SE from three measurements on different plants by using a radiogasometric method (Pärnik and Keerberg, 2007). All other values were calculated as means ±SE by multi-component non-linear regression analysis from the time-course of ^14^C-incorporation (simultaneous fit to equations 1–4; labelling data from three independent experiments).

	Carbon fluxes	*F. cronquistii* µmol m^–2^ s^–1^	% *P*_T_	*F. pubescens* µmol m^–2^ s^–1^	% *P*_T_	*F. trinervia* µmol m^–2^ s^–1^	% *P*_T_
*P*_T_*	True photosynthesis	3.76±0.10		7.93±0.70		10.37±0.28	
*R*_1_	CO_2_ incorporation directly into RPPC	3.82±0.49	101.6	6.23±0.07	78.6	0.45±0.25	4.3
*R*_6_	CO_2_ incorporation directly into C_4_ acids	0.32±0.01	8.5	1.29±0.32	16.3	9.42±0.10	90.8
*R*_7_	Secondary labelling of C_4_ acids C_1_–C_2_–C_3_	0.43±0.07	11.4	1.71±0.03	21.6	1.76±0.21	17.0
*R*_8_	C_4_ acid immobilization as end-products	0.10±0.01	2.7	0.66±0.06	8.3	0.94±0.15	9.1
*R*_1_+*R*_6_	Total CO_2_ incorporation	4.14±0.49	110.1	7.52±0.33	94.8	9.87±0.27	95.2
	*of which sucrose formation amounts to	0.95±0.02	25.3	2.11±0.09	26.6	5.98±0.63	57.7
	*of which starch formation amounts to	0.86±0.02	22.9	1.58±0.13	19.9	1.80±0.12	17.4
	*of which insoluble material amounts to	0.55±0.02	14.6	0.75±0.02	9.5	1.62±0.30	15.6
*R*_2_	C flow through photorespiratory pathway	6.64±0.25	176.6	3.66±0.20	46.2	2.56±0.21	24.7
*R*_2_/4	Decarboxylation of glycine	1.66±0.06	44.1	0.92±0.05	11.6	0.64±0.05	6.2
DEC*	Photorespiratory and C_4_ decarboxylation	2.18±0.08	58.0	1.84±0.05	23.2	6.70±0.21	64.6
*R*_P_*	Photorespiratory CO_2_ evolution	1.35±0.05	35.9	0.16±0.02	2.0	0.03±0.02	0.3
D*	Reassimilation in % of DEC		38.1	91.3		99.5	
*R*_2_/2	Oxygenation	3.3		1.8		1.3	
*R*_1_+*R*_6_+D*R_2_/4	Carboxylation	4.8		8.3		10.5	
	Mean relative CO_2_ at Rubisco sites	1.0		3.2		5.7	

CO_2_ can become incorporated into the RPPC either directly with rate *R*_1_ or indirectly via the C_4_ pathway with rate *R*_6_. The sums *R*_1_+*R*_6_ then represent total CO_2_ incorporation from external sources and show an increasing contribution by the C_4_ cycle, very low in *F. cronquistii*, low in *F. pubescens*, and, as expected, very high in *F. trinervia*. These total influx rates correspond reasonably well to directly measured rates for true photosynthesis *P*_T_, which provides a strong argument for the soundness of all other flux calculations. Higher values for *P*_T_ (C_3_<C_3_–C_4_<C_4_) go together with increased rates of sucrose formation (*R*_5_; directly measured in Table 1) and C_4_ acid accumulation as end-products (*R*_8_). Moreover, the photosynthetically active pools of C_4_ acids (*C*_7_; not listed in Table 1) increased from 13±1 (C_3_) via 57±19 (C_3_–C_4_) to 161±39 µmol C m^–2^ (C_4_). It is important to note that the increase of C_4_ cycle activity from *F. cronquistii* to *F. pubescens* (5.8% to 8.3% of *P*_T_, calculated as R_6_–R_8_) is only very small in comparison with the activity of the C_4_ cycle in *F. trinervia* (81.7% of *P*_T_). This suggests that CO_2_ accumulation occurs mainly by glycine-shuttling and less by C_4_ cycle activity in the bundle sheath of *F. pubescens*.

Carbon flux through the glycolate cycle, *R*_2_, is stoichiometrically related to the rate of RuBP oxygenation, *R*_2_/2. As a result of the operation of CO_2_-concentrating mechanisms in *F. pubescens* and in *F. trinervia*, photorespiration-related fluxes become distinctly lower from C_3_ towards C_4_ metabolism. To determine the true rates of RuBP carboxylation, in addition to the sum of *R*_1_ and *R*_6_, it was necessary to consider the refixation of CO_2_ generated from internal sources. In C_3_ and C_3_–C_4_ plants, photorespiration is the dominating internal source of CO_2_ during photosynthesis. *R*_2_ is stoichiometrically related to photorespiratory glycine decarboxylation as *R*_2_/4, because one molecule of CO_2_ is released per one molecule of serine formed from two glycine molecules. The extent to which refixation occurs must be separately determined. This was done by radiogasometric measurements (Pärnik and Keerberg, 1995, 2007), which allowed direct quantification of the sum DEC of photorespiratory glycine decarboxylation plus C_4_ acid decarboxylation plus minor CO_2_ releasing processes. It is reasonable to assume that all fractions of internally generated CO_2_ are re-assimilated with the same efficiency. In combination with the rate *R*_P_ of CO_2_ losses from the leaf (simplifying referred to as photorespiratory CO_2_ evolution), this assumption allows assessing the partitioning *D* between re-fixation and loss of CO_2_ from the leaf. The calculated total rates with which Rubisco fixes CO_2_ arriving by diffusion from the stomata (*R*_1_), from decarboxylation in the C_4_ cycle (*R*_6_), and from photorespiration (D*R_2_/4) were related to RuBP oxygenation rates (*R*_2_/2). The comparison shows that the resulting *in vivo* carboxylation-to-oxygenation ratio of Rubisco is more than three times higher in *F. pubescens* relative to *F. cronquistii* under the same experimental conditions.

Rubisco from C_4_ *Flaveria* species has a somewhat lower affinity to CO_2_, but it is also known that Rubisco from C_3_ and C_3_–C_4_ *Flaveria* species show more or less identical kinetics (Bauwe, 1984;Wessinger *et al.*, 1989; Kubien *et al.*, 2008). Since the oxygen compensation point of C_3_ plants is only slightly above air levels (Tolbert *et al.*, 1995), plastidial oxygen concentrations are probably close to air oxygen concentrations in *F. cronquistii* and *F. pubescens* but presumably also in the C_4_ species *F. trinervia*. Therefore, in a comparison of these species, measurement of *in vivo* carboxylation-to-oxygenation ratios allows the calculation of the relative CO_2_ concentration in chloroplasts. Considering the reported *K*_m_ values of Rubisco for CO_2_, which are even somewhat higher than steady-state internal CO_2_ levels, our data suggest that the photorespiratory CO_2_ pump elevates the mean intraplastidial CO_2_ concentration during steady-state photosynthesis about 3-fold in leaves of the C_3_–C_4_ intermediate species *F. pubescens* relative to the C_3_ species *F. cronquistii*. This is considered to be a sound estimate because small contributions from C_4_ photosynthesis are balanced by the operation of a significant fraction of Rubisco at non-elevated CO_2_ levels in the mesophyll of *F. pubescens.*

## Supplementary data

Supplementary data can be found at *JXB* online.

Supplementary data. An explanation of the labelling functions of the model shown in Fig. 1 used for the quantitative analysis of the labelling kinetics.

Supplementary Data

## Abbreviations:

CAM: crassulacean acid metabolism
GDC: glycine decarboxylase
RPPC: reductive pentose phosphate cycle (Calvin–Benson cycle)
RubP: ribulose 1,5-*bis*phosphate.
