# Supplementary material for: C2 photosynthesis generates about 3-fold elevated leaf CO2 levels in the C3–C4 intermediate species Flaveria pubescens
Source: J Exp Bot. 2014 Jun 10;65(13):3649–56. doi: 10.1093/jxb/eru239 (PMC4085972; doi:10.1093/jxb/eru239)
Supplement: Supplementary Data [file supp_65_13_3649__index.html]

C2 photosynthesis generates about 3-fold elevated leaf CO2 levels in the C3–C4 intermediate species Flaveria pubescens — C2 photosynthesis generates about 3-fold elevated leaf CO2 levels in the C3–C4 intermediate species Flaveria pubescens — Supplementary Data 

# C2 photosynthesis generates about 3-fold elevated leaf CO2 levels in the C3–C4 intermediate species *Flaveria pubescens*

## Supplementary Data

Data files

**Files in this Data Supplement:**

- Supplementary Data - Supplementary Data
