# Supplementary material for: C2 photosynthesis generates about 3-fold elevated leaf CO2 levels in the C3–C4 intermediate species Flaveria pubescens
Source: J Exp Bot. 2014 Jun 10;65(13):3649–56. doi: 10.1093/jxb/eru239 (PMC4085972; doi:10.1093/jxb/eru239)
Supplement: Supplementary Data [file supp_eru239_MS_Supplementary_Data.pdf]

Supplementary Data

Pool labeling functions

The time dependence of radioactivity of model components when $^{14}CO_2$ is applied under steady-state conditions is described by the following four functions

$$P(SP) = S_S\left[C_1E_A(t, C_1, R_S) + C_6E_I(t, C_1, C_6, R_5)\right],$$

$$P(Gly) = S_S\left[C_2E_I(t, V_1, C_2, R_2) + C_4E_I(t, V_2, C_4, R_3)\right],$$

$$P(Ser) = S_S\begin{bmatrix} C_3E_I(t, V_4, C_3, 0.75(R_2 - R_3)) + C_5E_I(t, V_6, C_5, R_4) + \\ V_6E_I(t, V_3, V_6, 0.75R_3) \end{bmatrix},$$

$$P(C4) = 0.25S_CC_7E_A(t, 0.25C_7, R_C) + S_S\begin{bmatrix} 0.75C_7E_I(t, V_7, 0.75C_7, R_7) + \\ E_E(t, V_8, 0.25R_8) + \\ E_E(t, V_9, 0.75R_8) \end{bmatrix},$$

where E_A, E_I and E_E are labeling functions described elsewhere (equations 1-3 in Keerberg *et al.*, 2011).

$$V_1 = R_2(C_1 + C_6)/R_S$$

$$V_2 = R_3(V_1 + C_2)/R_2$$

are the precursor pools for different components of the glycine branch of the photorespiratory pathway,

$$V_3 = 3(V_2 + C_4)/4$$

$$V_4 = 3(R_2 - R_3)(V_1 + C_2)/4R_2$$

$$V_5 = 4R_4(V_4 + C_3)/3(R_2 - R_3)$$

$$V_6 = C_1R_5/R_S$$

are the precursor pools for different components of the serine branch of the photorespiratory pathway,,

$$V_7 = R_7(C_1 + C_6)/R_S$$

$$V_8 = 0.25R_8C_7/4R_C$$

$$V_9 = 2.25R_8(V_7 + 0{,}75C_7)/4R_7$$

are the precursor pools for different components of the total C_4 acid pool, and

$$A_1 = R_6/(R_1 + R_6)$$

$$A_2 = R_1/(R_1 + R_6)$$

are the partition coefficients describing distribution of refixed photorespiratory CO_2 between the RPPC and the C_4 photosynthetic cycle.

Rates of carboxylation of RuBP in the RPPC, R_S, and carboxylation of PEP in the C_4 cycle, R_C, are described by

$$R_S = R_1 + R_6 + R_2(D + 3)/4$$

$$R_C = R_6 + A_1DR_2/4.$$

At the start of experiment, the specific radioactivity of CO_2 fixed in the RPPC, S_S, and in the C_4 cycle, S_C, are not equal to the specific radioactivity of $^{14}CO_2$ fed to leaves. They are diluted by carbon derived from non-labeled intermediates of the glycolate cycle according to the formula

$$S_S= [R_1 + R_6E_A(t,0.25C_7,R_C)]/[R_S – B_1 – B_2E_A(t,0.25C_7,R_C)]$$

$$S_C = [R_6 + S_SE_A(t,0.25C_7, R_C)]/R_C,$$

where

$$B_1 = [0.75(R_2 – R_3) –R_4]E_l(t,V_2,C_3,0.75(R_2 – R_3)) +$$

$$+ R_4E_l(t,V_5,C_5,R_4) + 0.75R_3E_l(t,V_3,V_{10},0.75R_3) +$$

$$+ 0.25DA_2[(R_2 – R_3)E_l(t,V_1,C_2,R_2) + R_3E_l(t, V_2,C_4,R3)]$$

$$B_2 = 0.25DA1[R_3E_l(t,V_2,C_4,R_3) + (R_2 – R_3)E_l(t,V_1,C_2,R_2)]$$

$$V_{10} = R_3C_3(R_2 – R_3).$$
